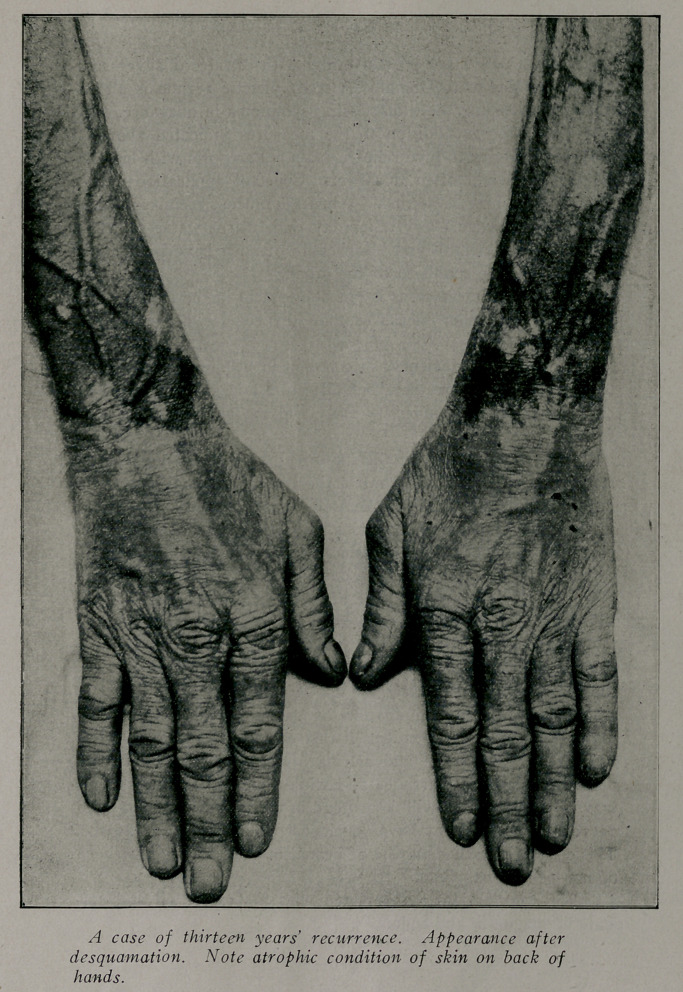# Etiology, Pathology and Treatment of Pellagra

**Published:** 1912-06

**Authors:** Geo. C. Mizell

**Affiliations:** Atlanta, Ga.; Gastro-Enterologist to Wesley Hospital; Formerly Associate Professor of Physiology and Gastro-Enterology of Atlanta College of Physicians and Surgeons


Journal-Record of Medicine

Successor to Atlanta Medical and Surgical Journal, Established 1855
and Southern Medical Record, Established 1870.
OWNED BY THE ATLANTA MEDICAL JOURNAL CO.
Published Monthly
*Official Organ Fulton County Medical Society, State Examining Board, Presbyterian Hospital, Atlanta, Birmingham and Atlantic Railroad Surgeons' Association, Chattahoochee Valley Medical and Surgical Association, Etc.*

EDGAR G. BALLENGER., M. D., Editor.
BERNARD WOLFF, M. D., Supervising Editor.
A. W. STIRLING, M. D., C. M., D. P. H., J. S. HURT, B. Ph., M. D.
GEO. M. NILES, M. D., W. J. LOVE, M. D., (Ala.); Associate Editors.
E. W. ALLEN, Business Manager.
COLLABORATORS
Dr. W. F. WESTMORLAND, General Surgery.
F. W. McRAE, M. D., Abdominal Surgery.
H. F. HARRIS, M. D., Pathology and Bacteriology.
E. B. BLOCK, M. D., Diseases of the Nervous System.
MICHAEL HOKE, M. D., Orthopedic Surgery.
CYRUS W. STRICKLER, M. D., Legal Medicine and Medical Legislation.
E. C. DAVIS, A. B., M. D., Obstetrics.
E. G. JONES, A. B., M. D., Gynecology.
R. T. DORSEY, Jr., B. S. M. D., Medicine.
L. M. GAINES, A. B., M. D., Internal Medicine.
GEO. C. MIZELL, M. D., Diseases of the Stomach and Intestines.
L. B. CLARKE, M. D., Pediatrics.
EDGAR PAULIN, M. D., Opsonic Medicine.
THEODORE TOEPEL, M. D., Mechano Therapy.
R. R. DALY, M. D., Medical Society.
A. W. STIRLING, M. D., etc., Diseases of the Eye, Ear, Nose and Throat.
BERNARD WOLFF, M. D., Diseases of the Skin.
E. G. BALLENGER, M. D., Diseases of the Genito-Urinary Organs.

VOL. LIX. JUNE 1912 NO. 3

### ETIOLOGY, PATHOLOGY AND TREATMENT OF PELLAGRA.

BY GEO. C. MIZELL, M. D.
*Gastro-Enterologist to Wesley Hospital; Formerly Associate Professor of Physiology and Gastro-Enterology of Atlanta College of Physicians and Surgeons.*

*(Continued from Last Issue.)*

Continuing the discussion of the eleven expressed objections as set forth by the Secretary of the State Board of Health, we come now to Objection Nine, which is as follows:

(9 "It has never been shown that even rancid cotton seed oil or other oils taken from wholesome sources are in any way poisonous. The statement of Lombroso that he found a poison in corn oil, from which the cotton seed oil theory probably had its origin, has been more or less disputed; and it should be remembered that this oil was taken from fermented corn, and there is every reason to believe that it was not in a pure state. It is quite conceivable that some of the poisonous substances derived from the corn might have been dissolved in the oil, since no attempt was made by Lombroso to free it from its impurities."

Since cotton seed have been shown to be deleterious to health of animals it must be concluded that cotton seed oil is not derived from an altogether wholesome source.

The Secretary is correct in stating that it has never been shown that even rancid cotton seed oil (or any other oil of this class) is any way poisonous. However, we know that rancid fat is injurious to the digestive mucosa. It can also be stated that there has never been any work done to show that cotton seed oil is a wholesome food. Some discussion of the probable relation of corn oil to pellagra will be found in Journal-Record of May, 1912. Pellagra, by Marie, translated by Lavinder and Babcock, goes thoroughly into the chemistry of spoiled corn. The suggestion that the cotton seed oil theory probably had its origin in the statement of Lombroso that he found a poison in corn oil is not correct. The first thing that directed my attention to cotton seed oil as a probable cause of pellagra was that investigation developed the fact that every case of pellagra was a consumer of cotton seed oil products and that not every pellagrin was a consumer of corn products.

(10) "The poisonous effects of cotton seed have been shown by the result of substances in no way related to its oil, and it is definitely known that these substances are entirely removed from cotton seed oil before it is offered for sale in the open market."

It is true that small quantities of cholin and betain have been isolated from cotton seed. These substances are poisonous, but it has not been shown that they are present in poisonous amounts. Cholin is a normal constituent of nerve tissue. Betain is a decomposition product of cholin.

The following is from Cottonseed Products, by Lamborn:

"Nearly all of the carefully conducted experiments show that neither cotton seed nor meal can be fed profitably to hogs and young calves. Not only can they not be fed profitably, but generally they are positively injurious and most frequently result in the death of the animal when persisted in. Whether death in these classes of animals is due to such mechanical causes as loose lint, large amount of oil, hard and sharp seed-coats, or whether cotton seed products contain originally a toxic principal, or whether such is developed as the result of decomposition outside of or change within the animal body, is yet an open question, and is a nice and important one, to be solved in connection with the problem of feeding cotton seed products.

"*Toxic Principles of Cotton Seed Meal.*—The extensive use of cotton seed meal for feeding purposes and the experience that it cannot be fed with safety to very young animals have directed attention to the occurrence in cotton seed meal of bodies possessing toxic properties. Chemical analysis has isolated two nitrogenous bases termed cholin and betain, the latter being a product of the oxidation of the former and the less poisonous of the two. In a sample of cotton seed cake examined in the U. S. Government laboratory the two bases were found present in the following relative proportion: viz., cholin 17.5 per cent., betain 82.5 per cent. Where toxic results have been experienced it is presumed that in the feed used, cholin has been relatively more abundant than betain.")

It is well known that cotton seed meal and hulls are much more wholesome for cattle than the whole seed and this fact would suggest that the oil is the injurious agent. Cotton seed meal and hulls in the proportion fed to stock contain less than five per cent. of oil, while the whole seed contain sixteen to twenty per cent.

As to the removal of the poisonous substances from the oil before it is offered for sale, it may be said that as far as wholesomeness is concerned the oil direct from the seed, without refining, is consumed with as much impunity as refined oil. The aim in refining is to render the oil palatable and not to remove any poisonous substance.

After stating this much to show that there is no basis for Objection Ten, it may be added that this objection, even if true, could have no weight against the cotton seed oil theory except as it affects the significance of the results when cotton seed products are fed to animals.

Nearly all vegetable and animal matter contain poison in only a small amount. Most vegetables which enter into our daily diet contain poisons, yet no evil effects are produced Lamborn expresses the true status of the question.

(11) "Pellagra prevails to a frightful extent in Egypt and in other portions of Africa, where the peasants eat no oil of any sort, and practically subsist on corn products."

In offering this objection, the Secretary shows again that his idea of the oil theory is not what the writer has tried to convey in these articles. In several places in these pages the possibility of corn producing pellagra has been insisted on. If it is true that any people anywhere practically subsist on corn products alone and develop pellagra as the result, it is not out of harmony with oil theory. This objection may be as incorrect as most of the others, for in Africa oleaginous seed abound and it would be remarkable if the natives have not followed the custom that prevails in other such regions. In fact, the authorities state that these oils are consumed in their respective homes. A list of these oil-producing seed, together with their nativity, as compiled from standard works, has been given in an earlier article.

### Treatment.

If the above conception of the disease is correct, it becomes apparent that there is no specific in the sense that quinine is a specific for malaria or antitoxin for diphtheria. All that any management can hope to accomplish here is to protect the tissues and reduce the sequellae to the minimum. Unlike all other diseases, pellagra is a condition in which the more slowly the primary cause is eradicated the less damage is done. Time is required for the removal of the unstable constituents of the tissues which seem to serve normal functions fairly well under some conditions. These conditions appear to be in the nature of influences favoring slow oxidation. This is in evidence in the beneficial effects of mental and physical rest, protection from sunshine and in the evil effects of incidents which call upon the reserved fuel of the body.

By respecting these views, the "peculiar exacerbations" by which pellagra is characterized may be avoided. The treatment to be outlined is especially directed to the relief of these exacerbations but when these are relieved much remains to be done before these patients are out of danger. The course of the disease in the exacerbation and following them can be greatly influenced, yet, not unlike other diseases, much irreparable damage may occur even before the diagnosis is made.

In mild, uncomplicated cases, scarcely any treatment is needed, but as it cannot be foretold when a mild case will develop serious symptoms, they should all be guided by certain general rules. The hygienic management should, of course, embrace the general requirements conducive to good health, such as plain, simple, nutritious food, regular hours and sufficient rest. When circumstances permit, outdoor exercises should be taken before eight o'clock in the morning and after five in the afternoon on days when the sun is shining. Exercise is always to be moderate —never enough to fatigue. Calcium Sulphid in ½ grain doses before meals should be given.

It is not often that mild, uncomplicated cases of pellagra present themselves for treatment, hence in all cases a thorough examination of the vital organs, secretions and excretions should be made. The generative organs in the female should be carefully examined. No simple deviation from the normal should go without attention, for, bear in mind, these patients are very susceptible to direct and reflex influences. For the sake of the digestive tract, do not overlook the teeth. One exception to this general overhauling is that until the pellagrous symptoms have passed off, no attention should be given to defects in vision as they are often transitory.

*Dietetic Treatment.*—For obvious reasons, all cereals containing much vegetable oil should be interdicted. Among these may be mentioned corn products and oatmeal. Mild, uncomplicated cases, who have not had pellagrous diarrhoea, may be given any plain, nutritious foods, avoiding fried foods, acids and concentrated sweets. They may also have grapes, oranges and peaches in limited amounts. They should have as much food and as varied a diet as the condition of the stomach and bowels will permit.

Meats are usually better borne than any class of foods, as a whole, and should be given twice a day. When stomatitis renders chewing painful, the meats should be well selected, tender, and free from connective tissue, and scraped or ground fine. Thus prepared, broiled and fed with a few crackers or toast, it is not only not irritating but has a marked beneficial effect on the sore mouth. Of the meats, roasted and broiled beef, mutton and chicken take first place. Except in unusual cases, where diarrhoea seems to be unfavorably influenced by meats, they should be depended on in all serious phases of pellagra.

The physician in pellagrous regions will often be confronted by cases who present the following symptoms: persistent nausea, vomiting, salivation and stomatitis and diarrhoea or constipation. Also there may be very feeble heart action.

This combination of symptoms is always of serious import and prompt, cautious action is necessary. Diet here is of the first importance and after experience with a large number of cases, the following directions are insisted on: When the circulation is good, a glass of sweet milk, to which an ounce of lime water has been added, and two or three crackers are given every two and one-half hours. In some cases when the milk is not retained, or the circulation is weak, solid food, viz., broiled, scraped steak or chicken with crackers or toast should be given every two and one-half hours. During such attacks the water is limited to a glassful and given one-half with the solid food and one-fourth with medicine before eating and one-fourth with medicine after eating. Special emphasis should be given to chew the solid food well and resist all inclination to wash the food down with water. In controlling the nausea and vomiting, no agent is as effective as the solid food above indicated and when milk is not retained, is always resorted to. Often it is not an easy task to induce the patient to take nourishment, but by promising relief from the distressing symptoms, the physician will rarely fail in his efforts.

After nausea and vomiting have been controlled, special attention should be given to the diarrhoea if it persists. By far, the most successful diet in managing these symptoms consists of meat, medium done, with a small amount of toasted white bread or crackers. The food should be given at three hour intervals. Rarely the diarrhoea improves, only when milk in which a raw egg has been beaten is given. In other cases this symptom yields to treatment only when given a mixed diet of meat, eggs, milk and breads. Fats are to be prohibited except when constipation exists.

When there is no tendency to diarrhoea, the diet needs to be limited only by the gastro-intestinal problems of each individual case. In extending the diet, great care should be exercised to prevent a recurrence of the diarrhoea and this can best be done by adding one article of food at a time. Upon any indication of a relapse, the diet should at once be restricted by safe limitations. It is a great mistake to overfeed these patients and try to increase too rapidly their weight, or to try to stimulate a lagging appetite by active exercise.

The weakness in these patients is not often due to a lack of nourishment, but rather to quality of tissue which can be improved —not rapidly, but gradually. Only in mild cases and those who have had no pellagrous diarrhoea are buttermilk or fermented milk and fruits to be permitted. Alcoholics and predigested foods containing alcohol are never allowed in any case. This rule should never be deviated from.

*Drugs in Pellagra.*—Promiscuous drugging of all sorts is, as a rule, in the highest degree detrimental. Unless the proper management is at the same time instituted, experience will soon teach one to distrust drugs in pellagra. Perhaps improvement is due to the management rather than the various remedies which have recently received credit for giving relief. Among the remedies for which claims are now being made are the derivatives of arsenic—and it is not to be denied that the credit given arsenic is justified. A great many cases have, no doubt, been benefited by this remedy, together with the proper management—hygienic, dietetic and prophylactic. This much is conceded, at the same time there is no doubt in my mind as to the best remedy, and some investigation has led me to believe that others are getting equally good results by the same methods. Not only are good results apparent in the progress of the patient by relief of pellagrous symptoms, but these patients feel best when on the remedies outlined below.

Some to whose attention this treatment has been outlined have been skeptical. During last summer (1911) a physician (a personal friend) requested that he be shown some of the results claimed for the treatment. He was carrid to see two apparently hopeless cases. After his first visits he remarked, "One thing I have noticed and that is that the patients are satisfied, a state of mind that I have not seen before." He followed these cases for a month and was convinced that the progress made was up to representations.

All cases are placed upon 1-6 grain pills of calcium sulphid. This is usually given three pills before each meal. The number of pills may be increased to as many as twelve (in a capsule) at a dose, but rarely are more than six needed. When there is hyper- or normal acidity or where there is belching of sulphuretted hydrogen an enteric pill should be given. The physician should always be sure that the patient gets calcium sulphid, i. e., that the pill is soluble and that it has not *decomposed,* as it is prone to do. Never prescribe a pill containing more than a sixth of a grain or a tablet, as they are worthless. In nearly all cases, one of the three prescriptions given below is indicated.

When the mucous membranes are involved and there is acid in the gastric secretion up to or above normal give:

℞ Sodii Chlorat. ---------------------- ʒii

Aqua ----------------------------- ℥vi

M et ft. sol.

Sig.—Two teaspoonfuls in ¼ glass of water ½ hour after meals.

In hypo-acidity give:

℞ Sodii Chlorat. ______________________ ʒii
Acid Hydrochlor. _________________ ℥ss
Ess. Pepsin, qs. ____________________ ℥vi
M. et ft. sol.

Sig.—Two teaspoonfuls in ¼ glass of water ½ hour after meals. (Direct to take through a tube or rinse mouth with water in which a pinch of baking soda has been dissolved).

To stimulate appetite, tincture of nux vomica in ten minim doses may be given with either of the above. These prescriptions are given all cases with gastro-intestinal symptoms. However, there are times when they may be suspended for a day or two in order that more attention may be given to the administration of food and other remedies for nausea and vomiting.

Nausea and vomiting are sometimes the most obstinate of the serious symptoms and all efforts for relief should be instituted. Should these symptoms continue for twenty-four hours after beginning the above prescriptions and diet, leave off the medicines and administer

℞ Pulv. Acacia ____________________ ʒii

Bismuth Subcarb _________________ ℥ss

Calcium Carbonat. __________________ ʒi

M et ft. Chart XII.

Sig.—One powder in a little water before eating.

Should constipation exist, ten grains of calcined magnesia (light) may be added to the above. These measures persisted in will relieve the nausea and vomiting and after forty-eight hours the former treatment should be resumed and continued throughout the duration of pellagrous symptoms.

Some palliative treatment is often needed for the numerous complications which arise and although these complications may present classical features of an independent disease, it is often the case that they do not yield to recognized treatment for these conditions.

Surgical measures should rarely be instituted during an attack of pellagra, and more especially in a case where an attack is impending. Not infrequently has a serious attack been precipitated by operation for gall stones, ovariotomies, hysterectomies, when the condition for which the operation was performed was in reality a part of a pellagrous process. However, should the pellagra symptoms have been relieved for some months, the past attack is no bar to surgical measures. In fact, at this period, these patients appear to recover from operations rapidly.

As no treatment will give the best results without the proper consideration of the adjunct measures, those that have been of value will be given in detail.

*Skin.*—The cutaneous lesions of pellagra are self-limited and very little treatment is needed and it is inadvisable during the erythematous and inflammatory stages. Protection from sunlight is essential to comfort.

When the corium is exposed by desquamation and a serous exudate or abrasions predispose to infection or when crusts need to be softened, apply any bland ointment, as ointment of zinc oxid with ichthyol (5%). Keep the parts exposed to the drying influence of the air. Burning and stinging of the skin of the extremities sometimes yields to a warm or cold bath and a rub with powdered salt or lard.

These symptoms may subside only when calcium sulphid is pushed to the limit.

Exposure to strong incandescent light rays often suffices.

*Mouth.*—In salivation, as in many other symptoms, too much treatment is usually given. The mouth should be sponged or washed after each feeding with a solution of chlorate of soda, 1 to 100.

Pellagrous stomatitis is not an ulceration but is allied to the dermatitis in that the superficial epithelium is involved. Atropin, 1-150 to 1-200 grain has been given for the salivation, but its use has been almost abandoned as unnecessary and inadvisable. It should never be given when mental symptoms are present.

Stomatitis, accompanied by dryness of the mucous membrane, can be alleviated by spraying with liquid alboline and moistening frequently with cinnamon water.

Pilocarpine, 1-20 grain, should also be given at bedtime, or twice daily.

*A case of thirteen years' recurrence. Appearance after desquamation. Note atrophic condition of skin on back of hands.*

Rarely ulcers are present—mop with nitrate of silver solution, 40 grains to the ounce. A strict dietetic regime is all important in keeping these distressing symptoms in abeyance.

*Stomach.*—In addition to the measures indicated above, it may be necessary to give relief to pain. Paregoric with bicarbonate of soda will often tide over. Constant application of hot water bottles may be needed. When the mucous membranes permit, gastric lavage every other day with warm water to which subnitrate of bismuth and bicarbonate of soda have been added will improve the condition of the organ.

Pyrosis, in these cases is not due to acid in the stomach and yields without other measures than the regular pellagra treatment.

Muscular insufficiency, so common in pellagra, does not often give rise to gastrectacis, but dilatation and gastroptosis often calls for a suitable binder, to make walking patients more comfortable.

*Intestines.*—In by far the majority of cases—if not all—the status of the bowels will control the issue. In many cases, the intestines, although crippled themselves, are called upon to do the work of the stomach also. Pains may call for relief by hot applications, paregoric, and colon irrigation with a solution of bicarbonate of soda, on alternate days, with warm water to which a drachm of compound tincture of iodine has been added. Proctitis may be relieved by irrigation with the same solutions.

Deficient secretion of bile may play a part in diarrhoea—calomel may have to be given daily, a grain at bedtime. Rarely the diarrhoea persists after the above treatment is instituted in which case careful examination of gastric contents and feces may reveal some independent disease which is causing it. The usual remedies—opium and astringents, for diarrhoea have no place in pellagrous diarrhoea and while the frequent movements may be checked, they will return and in the meantime mental and nervous symptoms may have developed or have been aggravated. This class of remedies may be of benefit in complicating diarrhoea of non-pellagrous origin.

For constipation, the best remedies are castor oil and sulphate of magnesia and soda.

*Heart.*—If due attention is not paid to the circulation, some patients who seem to be progressing well, suddenly pass out, without giving so much as a sign of cardiac weakness. The nurse may find a lifeless patient who a few minutes before was bright, cheerful and apparently vigorous. Cardiac weakness is most liable to develop during diarrhoea and vomiting. Nitroglycerin, digitalis and strychnine should be given and with this spartein sulphate and adrenalin if needed to keep up force and tension. Anticipate rather than be too late.

Murmurs, related to anaemic murmurs, are often present even in cases with slight pellagrous symptoms—they do not add to the gravity of the situation.

Genito-urinary symptoms are often present in the female and are frequently distressing. Ovarian and uterine pain may demand administration of anodynes and local treatment. Vaginitis and vulvitis can be relieved by douches of the solution recommended for colic irrigation. For irritation of the external surfaces, apply vaseline night and morning, after a bath of cold water.

Painful and frequent urination is relieved by a few doses of acetate of potash and elixir of saw palmetto.

Insomnia is rare in a properly managed case. Sleeplessness is often the result of some discomfort. As sleep is greatly to be desired, pain should be relieved and hypnotics given. When pain is frequent, paregoric and a solution of veronal (4 grs.) each, a teaspoonful and repeated in one or two hours if needed. Veronal in solution is a harmless remedy for producing sleep in these patients.

*Typhoid Pellagra.*—No attacks of this type have developed in patients after they have been placed on the above treatment, however, limited experience does not warrant an opinion as to the best management. Although it is admitted that little can be done to relieve these cases, much may be done to prevent such a fatal state. Much stress has already been placed upon the influence of light and heat and further observation may show that these influences are the most common exciting causes of typhoid pellagra and other serious developments. It is not unusual for a turn for the worse to date from a move to the hospital or a few hours' exposure to strong daylight. Thus, in some of these manifestations there may be an analogy to heat stroke and sunstroke. The susceptibility of pellagrous tissue to light rays soon teaches the intelligent afflicted to seek the shade. However, before the lesson is learned, or acting on the advice of the medical attendant the stroke may fall. Well meaning, yet misguided, friends and relatives sometimes overcome the inclination of the victim to seek a dark corner.

Fortunately, some are beginning to believe that "God's sunshine," while beneficial properly applied, is fraught with harm in overdose.

The best method of dealing with "typhoid pellagra," then, is to prevent it. When developed, sustain the patient. Keep the temperature within harmless range by sponging—absolute quiet—and give the treatment as indicated above.

*Psychic Symptoms.*—These manifestations are not infrequently the most prominent symptoms. In many cases these symptoms are acute and transient, yet it is probable that every attack of pellagra does more or less irreparable damage to the cerebro-spinal system and to a certain degree this may be true of all the tissues. Should mental symptoms persist after the relief of the other active symptoms, then the case becomes one of insanity and should be managed accordingly.

As yet no marked mental or psychic symptoms have remained in patients who have persisted in the treatment outlined above. This is probably due as much to prophylaxis as to the treatment.

By prophylaxis here is meant the protection of the patient from the sun. For these symptoms no special treatment is given. Usually there is immediate improvement upon the administration of calcium sulphide. Hydrotherapeutic measures, when available, are valuable aid. They will have to be selected according to each individual case. Wet compresses applied to the limbs are grateful to those suffering with pains in the limbs. Extremes of temperature should be avoided during the presence of marked irritation, but good results may be obtained by using these measures after the pellagra symptoms have subsided. The same may be said of galvanic and faradic electricity. Vibratory and electric massage should be used in cases where there is tenderness along the spine and changes in the cord.

The physician who is conversant with the principles involved in the therapeutic and physiologic application of these measures, will recognize their field of usefulness in restoring stability to tissues that have been rendered feeble from any cause. Of great importance is the expressed attitude of the medical attendant in the presence of these patients. Being in general very susceptible to morbid impressions, the pellagrin should have continually impressed upon him the nature of the disease and the possibility of relief. He should also be assured that after twelve months, through treatment and prophylaxis, there will be no return of the disease, although he may experience suggestions through susceptibility to light influences of a recurrence.

401-3 Empire-Life Bldg.

## Figures and Tables

**Figure f1:**